# Point prevalence and clinical course of proteinuria in dogs with idiopathic non‐erosive immune‐mediated polyarthritis

**DOI:** 10.1111/jsap.13503

**Published:** 2022-05-04

**Authors:** L. Barker, S. McManus, S. Adamantos, V. Black

**Affiliations:** ^1^ Bristol Veterinary School University of Bristol Bristol BS40 5DU UK; ^2^ Paragon Veterinary Referrals Wakefield WF1 2DF UK

## Abstract

**Objectives:**

To describe the point prevalence and clinical course of proteinuria in dogs diagnosed with idiopathic non‐erosive immune‐mediated polyarthritis.

**Materials and Methods:**

Cases presenting to a single referral centre with a diagnosis of idiopathic non‐erosive immune‐mediated polyarthritis were retrospectively recruited from January 2009 to August 2018. Data including signalment, urinalysis, clinicopathological results, cytology from arthrocentesis, treatment and long‐term follow‐up were analysed. Dogs were defined as: non‐proteinuric (UPC <0.2), borderline proteinuric (UPC 0.2‐0.5) or overtly proteinuric (UPC >0.5).

**Results:**

Fifty‐eight dogs met the inclusion criteria. Twenty‐two dogs were overtly proteinuric (38%), eight dogs were borderline proteinuric (14%) and 28 dogs were non‐proteinuric (48%). Repeated urinalysis was performed in nine of 12 dogs with UPC greater than 2.0. The UPC decreased in all nine dogs, with the UPC decreasing to less than 0.5 in 44% of dogs. A greater than 50% decrease in UPC was noted in 44% of dogs, despite seven of nine (77%) receiving prednisolone as either monotherapy or in conjunction with an adjunctive immunosuppressive medication.

**Clinical Significance:**

Proteinuria was common in this cohort of dogs diagnosed with primary idiopathic non‐erosive immune‐mediated polyarthritis. The use of prednisolone does not appear to be contraindicated in proteinuric dogs with idiopathic non‐erosive immune‐mediated polyarthritis.

## INTRODUCTION

Proteinuria is defined as excessive loss of protein in the urine and is classified as prerenal, renal or postrenal. In previous studies, proteinuria has been reported to be associated with many underlying diseases including parasitic, viral, bacterial and neoplastic diseases. Proteinuria has been identified in several systemic inflammatory diseases including immune‐mediated haemolytic anaemia, pyometra, pleuritis, discospondylitis, urinary tract infections, diabetes mellitus and acute pancreatitis (Stull *et al*. 2008, Vaden *et al*. 2010, Al‐Ghazlat *et al*. 2011, Schaefer *et al*. 2011). The most accurate determinant of proteinuria is a calculation of 24‐hour urine protein loss however, urine protein:creatinine ratio (UPC) is an accurate representation of 24‐hour protein loss in dogs and has now become a surrogate marker for the diagnosis of protein‐losing renal disorders (Grauer *et al*. 1985, Morales *et al*. 2004, Xin *et al*. 2004).

In dogs, the International Renal Interest Society categorises proteinuria as non‐proteinuric (UPC <0.2), borderline proteinuric (UPC 0.2 to 0.5) and significantly proteinuric (UPC >0.5) (Lees *et al*. 2005, Brown *et al*. 2013). In addition, proteinuria is considered an important clinical finding and has been associated with disease progression and prognosis in chronic kidney disease (CKD) in both dogs and cats. Proteinuria is also associated with systemic hypertension and thromboembolic disease both of which can have a significant clinical and prognostic impact on patients (Jacob *et al*. 2005, Syme *et al*. 2006).

Immune‐mediated polyarthritis (IMPA) is a commonly diagnosed inflammatory disease in dogs, immune complex deposition is one of the possible causes of IMPA but autoimmunity has also been discussed as a potential cause (Clements *et al*. 2004). Presenting signs include pyrexia, stiffness and joint pain (Rondeau *et al*. 2005, Johnson & Mackin 2012) and treatment typically involves immunosuppressive medication. The diagnosis of IMPA is usually based on synovial joint fluid analysis, with the identification of non‐septic neutrophilic inflammation in multiple joints (Stone 2017).

In the human literature, polyarthritis is a complex disease process with multiple subcategories of disease identified, with variable prognoses, it is likely a similar process occurs in the canine patient. The combination of proteinuria and inflammatory polyarthritis is seen in people with systemic lupus erythematosus and rheumatoid arthritis, both of which are type III hypersensitivity reactions (Hochberg 1997, Majithia & Geraci 2007). These diseases are recognised in animals, although the diagnostic criteria are much less robust than in human medical practice (Chabanne *et al*. 1993).

Previous studies reported that between 1 and 10% of dogs with IMPA had proteinuria, with a UPC less than 1 in all cases (Jacques *et al*. 2002, Clements *et al*. 2004, Stull *et al*. 2008). In contrast with this, we considered that a larger proportion of dogs presenting to our clinic with IMPA had concurrent proteinuria and that in some of these cases it was severe (UPC >2).

This study aimed to report the prevalence of proteinuria in dogs with a first‐time diagnosis of idiopathic non‐erosive IMPA and to determine if proteinuria resolves on the treatment of the underlying IMPA.

## MATERIALS AND METHODS

### Case recruitment and data collection

The clinical database at a single referral hospital, between January 1, 2009 and August 31, 2018, was searched to identify dogs with a first‐time diagnosis of non‐erosive idiopathic IMPA. Ethical approval was achieved from the institutional ethical approval committee.

Medical records were examined and screened for a diagnosis of non‐erosive idiopathic IMPA based on imaging and clinicopathological results (synovial fluid analysis, infectious disease screening findings). Thoracic and abdominal imaging was performed using either thoracic radiography and abdominal ultrasound, or thoracic and abdominal CT scan. The distal limbs were imaged using either radiography or CT. If multiple limbs were clinically affected only one limb needed to be imaged for inclusion.

Dogs must have had urinalysis performed (urine sediment examination, dipstick, USG) during their initial screening to include UPC, these samples were either collected as free‐catch samples at the time of admission or ultrasound‐guided cystocentesis performed at the time of imaging for underlying triggers. The urinalysis and UPC were all performed by a single reference laboratory using Konelab 60i Prime (Thermo Fisher Scientific), urine protein was measured with the pyrogallol method and the urine creatinine with the enzymatic method. The UPC was characterised as per the ACVIM consensus statement as either non‐proteinuric (UPC <0.2), borderline proteinuric (0.2‐0.5) or overtly proteinuric (UPC >0.5) (Lees *et al*. 2005).

Data extracted from the medical records included duration of signs before diagnosis, immunosuppressive treatment given following diagnosis and long‐term follow‐up where available. The criterion for inclusion was a diagnosis of idiopathic non‐erosive IMPA; defined by an absence of triggers for IMPA via imaging (as described above). No animals could have changes on imaging deemed to be a trigger for their IMPA and no animals could have evidence of erosive disease on joint radiography, as reviewed by a board‐certified radiologist or resident in training supervised by a board‐certified radiologist. Dogs needed cytological evidence of neutrophilic inflammation (>10% neutrophils) of two or more joints, based on previous diagnostic criteria (Stone 2017) and needed serum biochemistry, concurrent urinalysis including UPC and inactive sediment examination to be performed at the time of diagnosis and before starting treatment. All cytology was reviewed by a board‐certified pathologist or resident in training supervised by a board‐certified pathologist. Vector‐borne infectious diseases testing was undertaken at the discretion of the attending clinician depending on geographical location, travel history, physical examination and clinical history.

Dogs were excluded if they had received prior treatment with prednisolone if long‐term case follow‐up for greater than 1 month after diagnosis was not available, if an underlying disease thought to be the trigger for the IMPA was found, if there was positive testing for known vector‐borne infectious causes of IMPA, or if there was an underlying condition which could adversely affect the urinalysis results (*e.g*. CKD, other causes of azotaemia, lower urinary tract signs or marked hyperproteinaemia, *e.g*. multiple myeloma).

### Outcome

Long‐term follow‐up to assess for recurrence of clinical signs was collected by contacting referring veterinary practices to review clinical history if there had been disease recurrence, this was defined as any combination of pyrexia, joint effusions, joint pain or lameness with a recorded need for escalation of prednisolone dose or the addition of adjunctive immunosuppressive therapy. Dogs were only assessed for recurring clinical signs after they had been discharged from the hospital.

### Statistical analysis

Data were entered into a commercial computer database (Microsoft Excel) and analysed using a commercial statistical package (SPSS statistics; v.25 IBM Corps). Data were assessed for normality, and descriptive statistics were calculated, including median and range for UPC results. The point prevalence of proteinuria was calculated, this was done by dividing the portion of dogs in each category (proteinuric, borderline proteinuric and non‐proteinuric) by the total patient population and then multiplying by 100 to represent a percentage.

## RESULTS

One hundred and fifty‐two dogs were identified with a diagnosis of idiopathic non‐erosive IMPA between January 2009 and August 2018. Medical records were reviewed and 68 (44.7%) of these dogs had concurrent urinalysis performed, 10 of these 68 dogs were further excluded due to concurrent disease affecting the diagnosis of idiopathic non‐erosive IMPA or which may affect the urinalysis results achieved (six with lower urinary tract signs, two with polyarthritis–meningitis syndrome, one with multiple myeloma and one with concurrent azotaemia).

Fifty‐eight dogs, therefore, met the inclusion criteria. Of these, there were five entire females, 21 neutered females, two entire males and 30 neutered males. Presenting breeds included cocker spaniel (n=5), Cavalier King Charles spaniel (n=4), Labrador retriever (n=4), whippet (n=4), springer spaniel (n=3), Chinese crested (n=2), corgi (n=2), Jack Russell terrier (n=2), English pointer (n=2), and 13 dogs of mixed breeds and one each of 18 other breeds. The median age at the initial presentation was 5 years 1 month (range 8 months to 10 years 10 months).

Presenting clinical signs included a combination of the following, pyrexia, lethargy, joint effusions, lameness and struggling to rise, consistent with previously described presenting signs for idiopathic non‐erosive IMPA. The duration of clinical signs before diagnosis ranged from 1 to 632 days. Dogs had evidence of non‐septic neutrophilic inflammation in multiple joints, ranging from 19 to 99% of the total nucleated cells identified on synovial fluid cytology.

### Urinalysis

Cytological examination of the urine sediment was performed in all cases and was unremarkable in all dogs, for those cases which had a urine culture performed this was negative. The median UPC was 1.37 (range 0.01 to 8.86). Twenty‐two dogs (38%) were overtly proteinuric (UPC >0.5), eight dogs (14%) were borderline proteinuric (UPC 0.2 to 0.5), 28 dogs (48%) were non‐proteinuric (UPC <0.2). Twelve dogs (20%) had a UPC of greater than 2.

Of the dogs with mild to moderate proteinuria (UPC 0.5 to 2) nine of 10 had no follow‐up urinalysis performed, and one of 10 had repeated UPC and an improvement after initiating treatment for idiopathic non‐erosive IMPA with prednisolone monotherapy from 0.66 to 0.4, this was not deemed clinically significant. Of the dogs with marked proteinuria (UPC >2), three of 12 dogs had no follow‐up urinalysis performed, and nine of 12 dogs had a single repeated urinalysis which included UPC and sediment examination within 1 month of initiating treatment for idiopathic non‐erosive IMPA. All dogs had improvement in the UPC with four of nine dogs (44%) having normalisation of UPC to less than 0.5 and four of nine (44%) having a greater than 50% reduction in the UPC, one of nine dogs (11%) had persistent proteinuria which had decreased from 8.86 to 5.5, seven of nine of these dogs had improvement in UPC on treatment with either prednisolone monotherapy (n=3) or prednisolone and an adjunct treatment (n=4) (Table 1).

**Table 1:** Follow‐up UPC in dogs presenting with UPC greater than 2

Initial UPC	Follow‐up UPC	Treatment given
2.50	0.16	Prednisolone and leflunomide
2.63	ND	Prednisolone
2.78	ND	Prednisolone and leflunomide
2.97	ND	Prednisolone and azathioprine
3.28	1.28	Prednisolone
5.84	0.30	Prednisolone
6.70	0.10	Prednisolone and mycophenolate
6.72	3.26	Prednisolone
7.00	1.10	Leflunomide
7.10	0.40	Mycophenolate
7.28	1.06	Prednisolone and cyclosporine Aspirin
8.86	5.50	Prednisolone and cyclosporine Aspirin and benazepril

ND Not done

### Treatment

Initial immunosuppressive treatment given was prednisolone monotherapy (n=40) (dose range 1 to 4 mg/kg/day), prednisolone and leflunomide (n=4), prednisolone and cyclosporine (n=4), prednisolone and mycophenolate (n=3), prednisolone and azathioprine (n=2), mycophenolate (n=2) and leflunomide (n=2), one dog had dual treatment with cyclophosphamide and azathioprine. One proteinuric dog was started on benazepril and aspirin, and another proteinuric dog was started on aspirin, alongside the immunosuppressant medication, see Table 1. Dogs received additional analgesia as deemed appropriate by the clinician, including in‐hospital opioid therapy, and combinations of oral paracetamol, codeine, tramadol and gabapentin.

### Outcome

Follow‐up for cases was obtained by contacting referring veterinary surgeons to assess long‐term outcomes or recurrence of clinical signs for cases. For inclusion in the study animals needed a minimum of 1‐month follow‐up if alive, animals that had died had data collected at the time of death.

Thirty‐seven dogs (63%) were alive at the time of follow‐up, with a median follow‐up time of 720 days (range 37 to 2534 days). Twenty‐one dogs had died at the time of data collection with a median survival time of 350 days (range 22 to 1532 days), 52% of dogs were reported to be euthanised because of their disease, the euthanised animals had a median survival time of 130 days (range 22 to 1532 days). Contributing reasons included corticosteroid adverse effects, uncontrolled pain and recurrence of clinical signs of the disease.

Recurrence of disease was noted in 26 of 58 (44%) dogs, with a median time from diagnosis to recurrence of clinical signs of 178 days (range 30 to 1410 days). Reported clinical signs associated with recurrence of disease included stiffness/lameness (n=26), pyrexia (n=13) and joint effusions (n=8), in addition, lethargy was noted as a concurrent clinical sign in 14 of the dogs. All animals that were recorded to have a recurrence of their clinical signs had an escalation of immunosuppressive dose or addition of further immunosuppressive medications. Two dogs had repeated arthrocentesis at the time of recurrence of clinical signs and had neutrophilic inflammation present in multiple joints, consistent with a relapse of idiopathic non‐erosive IMPA.

Of all the dogs alive at the time of data collection, 28 of 37 (75%) were on ongoing long‐term immunosuppressive medication.

## DISCUSSION

Proteinuria was identified in 38% of dogs presenting to our hospital with idiopathic non‐erosive IMPA that had concurrent urinalysis performed during the study period, with marked proteinuria (UPC >2) identified in 20% of cases. These results contrast with previous studies which have identified only mild proteinuria (UPC <1) in 1 to 10% of cases of idiopathic non‐erosive IMPA in dogs (Jacques *et al*. 2002, Clements *et al*. 2004, Stull *et al*. 2008).

This study has revealed a higher prevalence of proteinuria at the time of admission than previously reported in dogs with idiopathic non‐erosive IMPA. The cause of proteinuria in these dogs is not fully understood; however, it is possible this could be a continuation of a type III hypersensitivity reaction and deposition of immune complexes within the nephrons causing glomerular nephropathy, the same pathogenesis as immune complexes forming within the joints in IMPA (Clements *et al*. 2004). Similarly, in human medicine, the cause of renal involvement and proteinuria in rheumatoid arthritis is unknown, with descriptions including it being due to adverse effects of drugs used for treatment, amyloidosis, vasculitis or unknown pathogenesis (Niederstadt *et al*. 1999, Koseki *et al*. 2001). Ultimately to determine the cause of proteinuria in these canine patients further investigation with renal biopsies would be needed.

Interestingly, three dogs in the study that had marked proteinuria (UPC >2), had complete resolution of the proteinuria and four dogs showed improving UPC despite treatment with prednisolone. Corticosteroids, including prednisolone, are known to contribute to proteinuria in dogs (Waters *et al*. 1997, Schellenberg *et al*. 2008), however, in this study marked proteinuria resolved despite corticosteroid use. Before this study, it would have seemed prudent to avoid prednisolone use in dogs with proteinuria and idiopathic non‐erosive IMPA, due to the concern of worsening the proteinuria. However, this study suggests that the use of corticosteroids is not associated with worsening proteinuria in dogs with idiopathic non‐erosive IMPA, and, based on their known efficacy for treatment (Rhoades *et al*. 2016) this makes them a sensible first choice immunosuppressive, however, due to the retrospective nature of this study, further prospective studies would be needed to confirm this hypothesis.

Survival in both dogs and cats with proteinuric CKD is known to be reduced compared to the survival of non‐proteinuric CKD patients (Jacob *et al*. 2005, Syme *et al*. 2006), and in dogs, with CKD a UPC of greater than 1 is associated with increased mortality (Jacob *et al*. 2005). Assessing survival was not the aim of this current study, however, the author finds it interesting to note that a proportion of the dogs with marked proteinuria responded well to treatment. Therefore the presence of proteinuria may not affect the outcome in idiopathic non‐erosive IMPA as it does in CKD, this interesting finding is beyond the scope of this paper and warrants exploration in future studies.

In human literature, polyarthritis is a well‐defined disease process, with many underlying diseases and triggers being identified including infectious and immune‐mediated diseases (Hoffman 1978, Majithia & Geraci 2007, Singh & Mehra 2010). Both SLE and rheumatoid arthritis cause non‐erosive and erosive polyarthritis, respectively, and present with concurrent proteinuria (Hochberg 1997, Majithia & Geraci 2007). The presence of proteinuria in non‐erosive IMPA was common in this study, but none of the dogs had ANA testing performed, therefore we cannot completely exclude that these findings could be consistent with SLE. As SLE is considered to be rare in dogs with a guarded prognosis and the proteinuric dogs in this study had a good long‐term prognosis, these cases are perhaps less likely to represent SLE (Bennett 1987a, Fournel *et al*. 1992). Due to the retrospective nature of the study, we cannot definitively determine if these dogs had SLE, however, the frequent occurrence of proteinuria in dogs with IMPA and the clinical course of this in response to treatment is still regarded as an interesting and important finding to report.

Overall the prognosis was moderate to fair for dogs in this study with those alive at the time of data collection having a median follow‐up time of 720 days. There is limited data in the current literature on the prognosis for dogs with idiopathic non‐erosive IMPA, with our study showing 15% of dogs were euthanised as a result of their disease, this is not dissimilar to what previous studies have identified (Bennett 1987b, Clements *et al*. 2004). Our study also shows similar rates of clinical response with 48% of patients experiencing a response to treatment of IMPA, compared to 56% (Clements *et al*. 2004) and 44% (Bennett 1987b) previously reported. Interestingly we noted a significant rate of recurrence of clinical signs (48%) in our study which is higher than the 20 to 31% previously reported (Bennett 1987b, Clements *et al*. 2004). These findings may represent variance in disease management, owners being more observant for evidence of recurrence of clinical signs, geographical variation or a genuine increase in recurrence of clinical signs from the current reports in the literature. The recurrence of disease including identification of stiffness/lameness by the attending veterinarian is a non‐specific finding, it cannot be excluded these were not steroid side effects (*e.g*. muscle weakness). Ultimately as relapse of the disease was not confirmed by repeated arthrocentesis or assessment and C‐reactive protein true relapse cannot be confirmed and is a recognised limitation of this study.

This study had many limitations, especially due to its retrospective nature. Firstly, this was a referral caseload and may not appropriately reflect the caseload of dogs with idiopathic non‐erosive IMPA presenting to primary care practices. In addition, not all dogs in this study had blood pressure measurements performed; as hypertension is associated with proteinuria in dogs we cannot exclude unidentified hypertension contributing to an increased risk of proteinuria in the study population. The treatment chosen for patients in this study was made by the attending clinician at the time of diagnosis, not all patients received the standard of care for proteinuria with RAAS inhibitors, antithrombotic or immunosuppression with mycophenolate as recommended by the consensus statement (Lees *et al*. 2005), the treatments given could therefore have affected the subsequent results. Finally, the proteinuria in these dogs was only noted on one urine sample, ideally, multiple UPCs should be documented to confirm proteinuria as per the ACVIM consensus statement, and therefore the effects of day to day variation in UPC could not be excluded (Nabity *et al*. 2007).

In conclusion, proteinuria was a common abnormality in dogs presenting to our hospital with idiopathic non‐erosive IMPA and appears to frequently resolve on the treatment of the underlying disease. Our results support screening for proteinuria as part of the diagnostic assessment of dogs presenting for idiopathic non‐erosive IMPA, as well as monitoring UPC on subsequent follow‐ups. Further prospective studies to assess the resolution of UPC when corticosteroids are used as a treatment for idiopathic non‐erosive IMPA, whether medical treatment for proteinuria is justified at initial diagnosis and also the use of UPC as a prognostic marker in dogs with idiopathic non‐erosive IMPA, are all areas which would benefit investigation in future studies.

### Conflict of interest

None of the authors of this article has a financial or personal relationship with other people or organisations that could inappropriately influence or bias the content of the paper.

## References

[jsap13503-bib-0001] Al‐Ghazlat, S. A. , Langston, C. E. , Greco, D. S. , *et al*. (2011) The prevalence of microalbuminuria and proteinuria in cats with diabetes mellitus. Topics in Companion Animal Medicine 26, 154‐157 21782146 10.1053/j.tcam.2011.04.005

[jsap13503-bib-0002] Bennett, D. (1987a) Immune‐based non‐erosive inflammatory joint disease of the dog. 1. Canine systemic lupus erythematosus. Journal of Small Animal Practice 28, 871‐889

[jsap13503-bib-0003] Bennett, D. (1987b) Immune‐based non‐erosive inflammatory joint disease of the dog. 3. Canine idiopathic polyarthritis. Journal of Small Animal Practice 28, 909‐928

[jsap13503-bib-0004] Brown, S. , Elliott, J. , Francey, T. , *et al*. (2013) Consensus recommendations for standard therapy of glomerular disease in dogs. Journal of Veterinary Internal Medicine 27(Suppl 1), S27‐S43 24635378 10.1111/jvim.12230

[jsap13503-bib-0005] Chabanne, L. , Fournel, C. , Faure, J. R. , *et al*. (1993) IgM and IgA rheumatoid factors in canine polyarthritis. Veterinary Immunology and Immunopathology 39, 365‐379 8116216 10.1016/0165-2427(93)90068-f

[jsap13503-bib-0006] Clements, D. N. , Gear, R. N. , Tattersall, J. , *et al*. (2004) Type I immune‐mediated polyarthritis in dogs: 39 cases (1997‐2002). Journal of the American Veterinary Medical Association 224, 1323‐1327 15112783 10.2460/javma.2004.224.1323

[jsap13503-bib-0007] Fournel, C. , Chabanne, L. , Caux, C. , *et al*. (1992) Canine systemic lupus erythematosus. I: a study of 75 cases. Lupus 1, 133‐139 1301973 10.1177/096120339200100303

[jsap13503-bib-0008] Grauer, G. F. , Thomas, C. B. & Eicker, S. W. (1985) Estimation of quantitative proteinuria in the dog, using the urine protein‐to‐creatinine ratio from a random, voided sample. American Journal of Veterinary Research 46, 2116‐2119 4062015

[jsap13503-bib-0009] Hochberg, M. C. (1997) Updating the American College of Rheumatology revised criteria for the classification of systemic lupus erythematosus. Arthritis and Rheumatism 40, 1725 10.1002/art.1780400928 9324032

[jsap13503-bib-0010] Hoffman, G. S. (1978) Polyarthritis: the differential diagnosis of rheumatoid arthritis. Seminars in Arthritis and Rheumatism 8, 115‐141 368983 10.1016/0049-0172(78)90015-x

[jsap13503-bib-0011] Jacob, F. , Polzin, D. J. , Osborne, C. A. , *et al*. (2005) Evaluation of the association between initial proteinuria and morbidity rate or death in dogs with naturally occurring chronic renal failure. Journal of the American Veterinary Medical Association 226, 393‐400 15702689 10.2460/javma.2005.226.393

[jsap13503-bib-0012] Jacques, D. , Cauzinille, L. , Bouvy, B. , *et al*. (2002) A retrospective study of 40 dogs with polyarthritis. Veterinary Surgery 31, 428‐434 12209413 10.1053/jvet.2002.34665

[jsap13503-bib-0013] Johnson, K. C. & Mackin, A. (2012) Canine immune‐mediated polyarthritis: part 1: pathophysiology. Journal of the American Animal Hospital Association 48, 12‐17 22190602 10.5326/JAAHA-MS-5744

[jsap13503-bib-0014] Koseki, Y. , Terai, C. , Moriguchi, M. , *et al*. (2001) A prospective study of renal disease in patients with early rheumatoid arthritis. Annals of the Rheumatic Diseases 60, 327‐331 11247860 10.1136/ard.60.4.327 PMC1753620

[jsap13503-bib-0015] Lees, G. E. , Brown, S. A. , Elliott, J. , *et al*. (2005) Assessment and management of proteinuria in dogs and cats: 2004 ACVIM forum consensus statement (small animal). Journal of Veterinary Internal Medicine 19, 377‐385 15954557 10.1892/0891-6640(2005)19[377:aamopi]2.0.co;2

[jsap13503-bib-0016] Majithia, V. & Geraci, S. A. (2007) Rheumatoid arthritis: diagnosis and management. The American Journal of Medicine 120, 936‐939 17976416 10.1016/j.amjmed.2007.04.005

[jsap13503-bib-0017] Morales, J. V. , Weber, R. , Wagner, M. B. , *et al*. (2004) Is morning urinary protein/creatinine ratio a reliable estimator of 24‐hour proteinuria in patients with glomerulonephritis and different levels of renal function? Journal of Nephrology 17, 666‐672 15593033

[jsap13503-bib-0018] Nabity, M. B. , Boggess, M. M. , Kashtan, C. E. , *et al*. (2007) Day‐to‐day variation of the urine protein: creatinine ratio in female dogs with stable glomeurular proteinuria caused by X‐linked hereditary nephropathy. Journal of Veterinary Internal Medicine 21, 425‐430 17552446 10.1892/0891-6640(2007)21[425:dvotup]2.0.co;2

[jsap13503-bib-0019] Niederstadt, C. , Happ, T. , Tatsis, E. , *et al*. (1999) Glomerular and tubular proteinuria as markers of nephropathy in rheumatoid arthritis. Rheumatology 38, 28‐33 10334679 10.1093/rheumatology/38.1.28

[jsap13503-bib-0020] Rhoades, A. C. , Vernau, W. , Kass, P. H. , *et al*. (2016) Comparison of the efficacy of prednisone and cyclosporine for treatment of dogs with primary immune‐mediated polyarthritis. Journal of the American Veterinary Medical Association 248, 395‐404 26829271 10.2460/javma.248.4.395

[jsap13503-bib-0021] Rondeau, M. P. , Walton, R. M. , Bissett, S. , *et al*. (2005) Suppurative, nonseptic polyarthropathy in dogs. Journal of Veterinary Internal Medicine 19, 654‐662 16231709 10.1892/0891-6640(2005)19[654:snpid]2.0.co;2

[jsap13503-bib-0022] Schaefer, H. , Kohn, B. , Schweigert, F. J. , *et al*. (2011) Quantitative and qualitative urine protein excretion in dogs with severe inflammatory response syndrome. Journal of Veterinary Internal Medicine 25, 1292‐1297 22092619 10.1111/j.1939-1676.2011.00829.x

[jsap13503-bib-0023] Schellenberg, S. , Mettler, M. , Gentilini, F. , *et al*. (2008) The effects of hydrocortisone on systemic arterial blood pressure and urinary protein excretion in dogs. Journal of Veterinary Internal Medicine 22, 273‐281 18312279 10.1111/j.1939-1676.2007.0039.x

[jsap13503-bib-0024] Singh, S. & Mehra, S. (2010) Approach to polyarthritis. Indian Journal of Pediatrics 77, 1005‐1010 20734164 10.1007/s12098-010-0163-5

[jsap13503-bib-0025] Stone, M. (2017) Immune mediated polyarthritis and other polyarthopathies. In: Textbook of Veterinary Internal Medicine. 8th edn. Eds S. J. Ettinger , E. C. Feldman and E. Cote . Elsevier, St. Louis, Missouri, USA. pp 861‐866

[jsap13503-bib-0026] Stull, J. W. , Evason, M. , Carr, A. P. , *et al*. (2008) Canine immune‐mediated polyarthritis: clinical and laboratory findings in 83 cases in western Canada (1991‐2001). The Canadian Veterinary Journal 49, 1195‐1203 19252711 PMC2583415

[jsap13503-bib-0027] Syme, H. M. , Markwell, P. J. , Pfeiffer, D. , *et al*. (2006) Survival of cats with naturally occurring chronic renal failure is related to severity of proteinuria. Journal of Veterinary Internal Medicine 20, 528‐535 16734085 10.1892/0891-6640(2006)20[528:socwno]2.0.co;2

[jsap13503-bib-0028] Vaden, S. L. , Turman, C. A. , Harris, T. L. , *et al*. (2010) The prevalence of albuminuria in dogs and cats in an ICU or recovering from anesthesia. Journal of Veterinary Emergency and Critical Care 20, 479‐487 20955298 10.1111/j.1476-4431.2010.00584.x

[jsap13503-bib-0029] Waters, C. B. , Adams, L. G. , Scott‐Moncrieff, J. C. , *et al*. (1997) Effects of glucocorticoid therapy on urine protein‐to‐creatinine ratios and renal morphology in dogs. Journal of Veterinary Internal Medicine 11, 172‐177 9183769 10.1111/j.1939-1676.1997.tb00086.x

[jsap13503-bib-0030] Xin, G. , Wang, M. , Jiao, L. L. , *et al*. (2004) Protein‐to‐creatinine ratio in spot urine samples as a predictor of quantitation of proteinuria. Clinica Chimica Acta 350, 35‐39 10.1016/j.cccn.2004.06.019 15530457

